# Oxidative Stress, a Crossroad between Rare Diseases and Neurodegeneration

**DOI:** 10.3390/antiox9040313

**Published:** 2020-04-15

**Authors:** Carmen Espinós, Máximo Ibo Galindo, María Adelaida García-Gimeno, José Santiago Ibáñez-Cabellos, Dolores Martínez-Rubio, José María Millán, Regina Rodrigo, Pascual Sanz, Marta Seco-Cervera, Teresa Sevilla, Andrea Tapia, Federico V. Pallardó

**Affiliations:** 1 Unit of Genetics and Genomics of Neuromuscular and Neurodegenerative Disorders, Centro de Investigación Príncipe Felipe (CIPF), 46012 Valencia, Spain; 2 Rare Diseases Joint Unit, CIPF-IIS La Fe, 46026 Valencia, Spain; 3 Rare Diseases Joint Unit, CIPF-INCLIVA, 46010 Valencia, Spain; 4 Instituto de Biomedicina de Valencia (IBV)-CSIC Associated Unit, Centro de Investigación Príncipe Felipe (CIPF), 46010 Valencia, Spain; 5 Instituto Interuniversitario de Investigación de Reconocimiento Molecular y Desarrollo Tecnológico (IDM), Universitat Politècnica de València, Universitat de València, 46022 Valencia, Spain; 6 Laboratory of Developmental Biology and Disease Models, UPV-CIPF Joint Research Unit Disease Mechanisms and Nanomedicine, Centro de Investigación Príncipe Felipe, 46012 Valencia, Spain; 7 Department of Biotechnology, Escuela Técnica Superior de Ingeniería Agronómica y del Medio Natural (ETSIAMN), Universitat Politècnica de València, 46022 Valencia, Spain; 8 Centre for Biomedical Research on Rare Diseases (CIBERER), Instituto de Salud Carlos III, 28029 Madrid, Spain; 9 Department of Physiology, Faculty of Medicine and Dentistry, Universitat de València (UV), 46010 Valencia, Spain; 10 Molecular, Cellular and Genomics Biomedicine Unit, IIS La Fe, 46026 Valencia, Spain; 11 Pathophysiology and Therapies for Vision Disorders Unit, Centro de Investigación Príncipe Felipe (CIPF), 46012 Valencia, Spain; 12 Nutrient-mediated Signaling Unit, Instituto de Biomedicina de Valencia-CSIC, 46010 Valencia, Spain; 13 Department of Neurology, Hospital Universitari i Politècnic La Fe, 46026 Valencia, Spain

**Keywords:** Friedreich’s ataxia, neurodegenerative disorders with brain iron accumulation (NBIA), Charcot-Marie-Tooth disease (CMT), inherited retinal dystrophy (IRD), progressive myoclonus epilepsy (PME), Unverricht–Lundborg disease (ULD), Lafora disease (LD), Dravet syndrome

## Abstract

Oxidative stress is an imbalance between production and accumulation of oxygen reactive species and/or reactive nitrogen species in cells and tissues, and the capacity of detoxifying these products, using enzymatic and non-enzymatic components, such as glutathione. Oxidative stress plays roles in several pathological processes in the nervous system, such as neurotoxicity, neuroinflammation, ischemic stroke, and neurodegeneration. The concepts of oxidative stress and rare diseases were formulated in the eighties, and since then, the link between them has not stopped growing. The present review aims to expand knowledge in the pathological processes associated with oxidative stress underlying some groups of rare diseases: Friedreich’s ataxia, diseases with neurodegeneration with brain iron accumulation, Charcot-Marie-Tooth as an example of rare neuromuscular disorders, inherited retinal dystrophies, progressive myoclonus epilepsies, and pediatric drug-resistant epilepsies. Despite the discrimination between cause and effect may not be easy on many occasions, all these conditions are Mendelian rare diseases that share oxidative stress as a common factor, and this may represent a potential target for therapies.

## 1. Introduction

Balanced levels of reactive oxygen species (ROS) and/or reactive nitrogen species (RNS) and antioxidant defense mechanisms are present in normal physiological processes. However, when this equilibrium is disturbed, a process called oxidative stress is observed. As a result of this imbalance cellular, damage is produced by excessive ROS/RNS [1].

Eukaryotic organisms need oxygen to normal physiological functions. Nevertheless, incomplete metabolic reduction of oxygen to water produces most cellular ROS. Reduction of oxygen forms radical superoxide (O_2_^•−^), which itself can produce hydrogen peroxide (H_2_O_2_). This molecule, in turn, may be fully reduced to inert H_2_O or may be partially reduced to hydroxyl radical (OH^•^) and hydroxide ion. One example of this partial reduction is Fenton reaction, where reduced metal ions, such as ferrous iron (Fe^2+^), act as a catalyst. ROS present elevated reactivity, which produces interactions with biological molecules and hence, changing normal cell functions that may lead to cell death. 

Sources of free radical species in humans can be divided based on their original source as internal or external. The main internal sources are mitochondria, peroxisomes, endoplasmic reticulum (ER), NADPH, and xanthine oxidases, ischemia-reperfusion mechanisms, and neutrophils, eosinophils, and macrophages activity. The main external factors are smoking and alcohol, infections, inflammatory processes, UV irradiation, photooxidation, electromagnetic radiation, and xenobiotics. In order to balance ROS content, cells promote antioxidant defenses mechanisms, which can be divided into enzymatic and non-enzymatic. The main non-enzymatic antioxidant in mammalian cells is glutathione (GSH), which has an essential role as a cofactor of different antioxidant enzymes, including glutathione peroxidase enzyme-4 (GPX4). This enzyme has a phospholipid hydroperoxidase activity that reduces lipid hydroperoxides (R–OOH) to lipid alcohols (R–OH). The activity of GPX4 needs two GSH molecules, which are converted to GSSG. GSSG can be recycled back to GSH in an NADPH-dependent manner by GSH reductase. In susceptible cells, inhibition of GPX4 [2,3] or GSH unavailability [4,5] triggers a lethal lipid peroxidation process.

Oxidative stress has been related to different pathological processes such as neurotoxicity, neuroinflammation, ischemic stroke, and neurodegenerative diseases, such as Parkinson’s disease, Alzheimer’s disease, Huntington’s disease, or Friedreich’s ataxia [6,7,8,9]. In this review, we focus in some groups of rare diseases: Friedreich’s ataxia, diseases with neurodegeneration with brain iron accumulation (NBIA), Charcot-Marie-Tooth as an example of rare neuromuscular disorders, inherited retinal dystrophies (IRDs), and progressive myoclonus epilepsies (PMEs) and pediatric drug-resistant epilepsies.

## 2. Friedreich’s Ataxia

Friedreich’s ataxia (FRDA, MIM 229300) is the most prevalent hereditary ataxia [10,11]. FRDA is a rare childhood-onset disease characterized by gait and limb ataxia, lower limb areflexia, and dysarthria. A mixed origin of ataxia results from spinocerebellar degeneration, peripheral sensory neuropathy, cerebellar, and vestibular pathology, and the posterior adding of the pyramidal disabilities [12]. Other non-neurological features of FRDA are scoliosis, diabetes, and cardiac symptoms [13,14,15]. The majority of FRDA patients are homozygous for an unstable guanine-adenine-adenine (GAA) expansion in the first intron of the *FXN* gene that localizes in chromosome 9q21.11 producing decreased protein levels of frataxin [16,17]. The principal function of frataxin is not clear; however, the early lethality in embryos of *Fxn* knockout mice underscores the importance of frataxin function in cell survival [18]. Previous studies have reported the involvement of the FXN protein in mitochondrial biogenesis [19] and the synthesis of iron-sulfur clusters (ISC) [20]. Mitochondrial respiratory chain dysfunction [21], mitochondrial iron accumulation [22], decreased mitochondrial DNA levels, oxidative stress [23,24], reduced generation of ATP [23], and altered lipid metabolism are molecular and pathological features of FRDA. One of the main pathways related to oxidative stress that is altered in FRDA is the Nfr2-pathway [25,26]. Human and mouse models of this disease showed a defective activation of phase II enzymes by Nfr2. Furthermore, different studies with Nfr2-inducers counteracts oxidative stress, cell death, and mitochondrial defects in different human and mouse models of FRDA [27,28].

**Figure 1:**
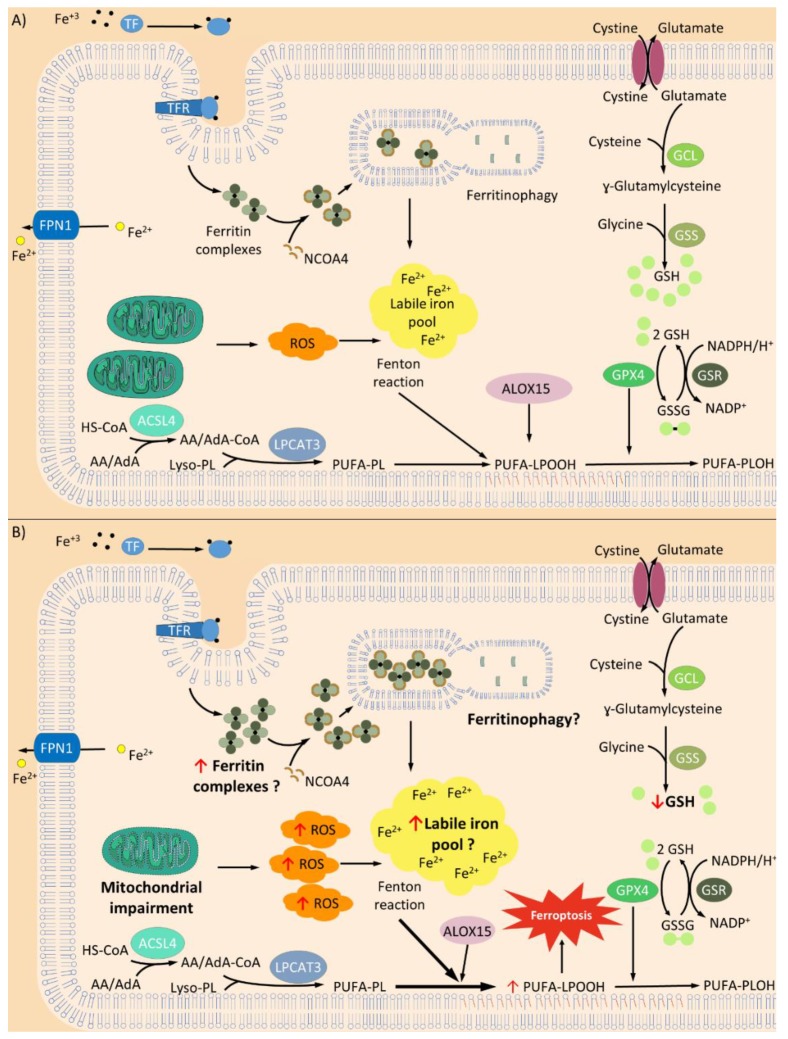
Main mechanisms in ferroptosis. (**A**) General process of ferroptosis. Balanced levels of reactive oxygen species (ROS) generation and antioxidant activity maintains an inactive ferroptosis cell death pathway; however, some disturbance in the players of this equilibrium could produce a ferroptosis activation. Two important components of ferroptosis are the process of transferrin (TF)-iron import and ferroportin (FPN1) export. Changes in iron uptake or iron export produce a suppression or activation of ferroptosis since the free iron pool is fundamental [31,32]. Ferroptosis may be enhanced through the process of ferritinophagy, by which ferritin is targeted by nuclear receptor coactivator 4 (NCOA4) and delivered to the lysosome for degradation in autophagosome [33,34,35]. Consequently, a large quantity of iron can be rapidly released. Currently, it has been suggested that ferroptosis initiation might be triggered by an increase in free iron levels itself [36]. Iron increase/or accumulation induces the Fenton reaction which increases the production of ROS. Furthermore, the action of iron-dependent oxidases [37], specifically lipoxygenase activity of 15-LOX (ALOX15) that oxidizes polyunsaturated fatty acids (PUFAs) phospholipids (PUFA-PLs) containing arachidonate or adrenate moieties, triggers the ferroptosis pathway [38,39]. In addition, increased activity of acyl-CoA synthetase long-chain family member 4 (ACSL4), an enzyme that preferentially activates arachidonic (AA) and adrenoic acids (AdA), has been reported to increase ferroptosis [38,40]. Inhibition of glutathione peroxidase enzyme 4 (GPX4) [2,3] or glutathione (GSH) unavailability [4,5] produces lipid hydroperoxide accumulation that triggers ferroptosis. (**B**) Suggested mechanisms of ferroptosis in Friedreich’s ataxia (FRDA). It has been observed increased levels of lipoperoxides and ROS in FRDA neurons. Furthermore, in these neurons, it has also been reported lower reduced GSH concentration and GSH homeostasis disturbance, respectively [41,42,43]. Controversial studies are reporting either normal or increased iron levels in the nervous system of FRDA patients (reviewed in [44]). Recently a study in FRDA patients, using magnetic resonance imaging, showed increased iron concentration in the extrapyramidal motor system compared with controls [45]. However, there is no information about distribution, location and redox state of the iron in cells from FRDA (indicated with “?”). Increased autophagic processes are related to FRDA in different models of disease [46,47], but there is no information about ferritinophagic activity. Further studies should be performed in order to unravel if this mechanism increases the iron content in FRDA cells. All these ferroptotic characteristics observed in FRDA may indicate that this kind of cell death may have a role in the physiopathology of this disease. Transferrin receptor (TFR) Lysophosphatidylcholine Acyltransferase 3 (LPCAT3); Glutamate Cysteine Ligase (GCL); Glutathione synthetase (GSS); Glutathione reductase (GR). ↑ indicates increased levels and ↓ indicates decreased levels.

Ferroptosis is a new term for regulated cell death pathways (RCD) that is remarkably distinct at morphological, biochemical, and genetic levels from other RCD, such as apoptosis, classical autophagy, and necrosis [5]. This pathway is characterized by the overwhelming, iron-dependent accumulation of lethal lipid hydroperoxides [5]. It is noteworthy that this RCD is not a novel concept and it has been described several times before [29,30]. Diverse players of the ferroptosis pathway have been reported in the literature as isolated effects, but it is still unknown their connections in the whole landscape of this RCD. Some of these players are shown in Figure 1. 

The discovery of ferroptosis as a possible way of neuronal death in FRDA is a major achievement since it explains many of the already known cellular, metabolic, and pathophysiological characteristics of neuronal degeneration in FRDA, like oxidative stress, changes in calcium levels, neuronal degeneration, and mitochondrial and membrane damage [48]. FRDA neurons showed higher lipoperoxide levels, increased ROS and lower reduced GSH concentration, and enhanced sensitivity to oxidants compared with control neurons [41,42]. Furthermore, patients with FRDA suffer a disturbance of GSH homeostasis (Figure 1) [43]. 

In recent years, a few studies have started to show the implication of miRNAs regulation in ferroptosis [49,50,51]. Additionally, small RNA analysis performed in FRDA revealed altered levels of miRNAs [52,53,54]. The plasma of FRDA patients showed higher levels of miR-130b-5p and miR-142-3p. Since these miRNAs target the mRNAs coding for proteins involved in fatty acid metabolism and β-oxidation, they could affect ferroptosis regulation [52]. All these results indicate that ferroptosis may be an important pathway linked with FRDA pathophysiology and hence, a potential therapeutic target.

## 3. Neurodegenerative Disorders with Brain Iron Accumulation

Syndromes of neurodegeneration with brain iron accumulation (NBIA) are a group of movement disorders, which share an abnormal deposit of iron in the brain, predominantly in the basal ganglia. NBIA disorders start at any age, from early childhood to adulthood, and the course is relentlessly progressive causing early death or severe motor, cognitive, and sensorial disabilities in survival patients. The clinical course includes progressive speech difficulties, dysphagia, dystonia, spasticity, parkinsonism, pure akinesia, ocular motor dysfunction, vision loss, and neuropsychiatric disturbances. NBIA forms are rare diseases with an estimated prevalence of less than 1/1,000,000.

**Figure 2:**
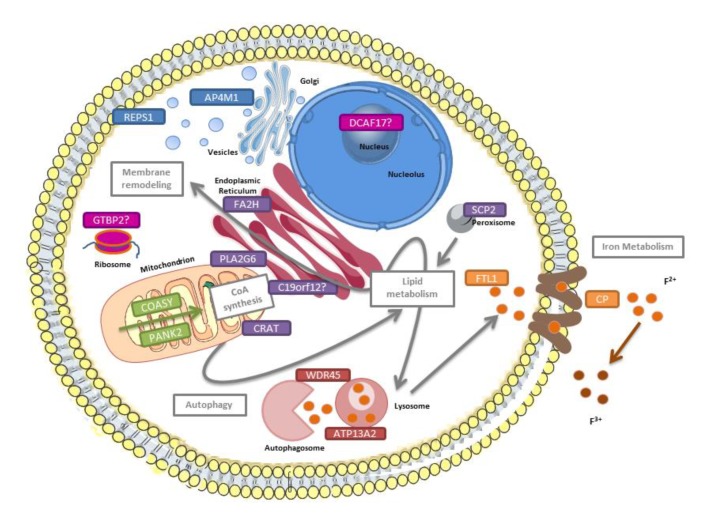
Pathways underlying NBIA disorders. *PANK2* and *COASY* (in green) are involved in the coenzyme A (CoA) synthesis. *PLA2G6*, *C19orf12*, *FA2H*, *SCP2*, and *CRAT* (in purple) are related to lipid metabolism and membrane remodeling. *CP* and *FTL1* (in orange) are implicated in iron metabolism; *WDR45* and *ATP13A2* (in maroon) play a role in autophagy; and finally, *REPS1* and *AP4M1* (in blue) are associated with vesicle trafficking. The function of *DCAF17* and *GTPBP2* (in fuchsia) remains elusive.

Ten NBIA genes are widely accepted, although, with the description of some cases, five additional genes could also be considered NBIA genes (Table 1) [55,56]. Little is known about the underlying disease mechanisms. Several pathways must be considered: iron and lipid metabolism, membrane remodeling, coenzyme A (CoA) synthesis, and autophagy (Figure 2). However, most of the genes play directly or indirectly a role in mitochondria and cause ion imbalance of Ca^2+^, mitochondrial-ER altered communication, and impaired oxidative phosphorylation (OXPHOS). In this review, the focus is on the NBIA forms in which altered ROS plays an essential role in the pathomechanism. 

*PANK2* and *COASY* are implicated in the biosynthesis of CoA. Mutations in *PANK2* cause PKAN (Pantothenate Kinase-Associated Neurodegeneration, MIM 234200), which is the most frequent NBIA form (35–50% of NBIA patients), and deficiency of COASY leads to COPAN (COasy Protein-Associated Neurodegeneration, MIM 615643), which is an ultra-rare form of NBIA (Table 1). PANK2 catalyzes the ATP-dependent phosphorylation of pantothenate, the first step of the CoA synthesis, whereas COASY regulates the last two steps in the CoA synthesis. In *Drosophila*, the dissection of the CoA synthesis route revealed that this pathway is important for maintaining DNA and cellular integrity [57]. Moreover, fly mutants for *Pank2* (*dPANK/fmbl*) and *Coasy* (*dPPAT-DPCK*), display locomotor dysfunction, increased sensitivity to oxidative stress, and altered lipid homeostasis [57].

The absence of PANK2 causes the accumulation of substrates in the CoA synthesis pathway, mainly *N*-pantothenyl cysteine and free cysteine. Free cysteine suffers spontaneous autoxidation due to the deposits of iron, which produces free radicals, and finally, oxidative damage and cell death [58,59]. The null fly model *fumble*, which is the orthologue of the human *PANK2* gene in *Drosophila melanogaster*, shows decreased CoA levels, mitochondrial dysfunction, increased protein oxidation and impairment of lipid homeostasis [60]. Neurons of the *Pank2* knockout mouse present altered mitochondrial membrane potential and defective respiration that would contribute to ROS generation [61]. In the murine *Pank2* model, signs such as motor dysfunction, neurological impairment, and premature death, are only observed when the mouse is fed with a ketogenic diet [62]. Fibroblasts and induced Pluripotent Stem Cells (iPSCs)-derived neurons, exhibit elevated levels of ROS production, together with premature death, and mitochondrial dysfunction, including impairment of mitochondrial iron-dependent biosynthesis, probably due to iron mishandling [63,64,65,66,67].

Mutations in *PLA2G6* cause PLAN (*PLA2G6*-associated neurodegeneration, MIM 615643). The encoded protein by *PLA2G6*, iPLA2-VI, hydrolyzes at the sn-2 position of glycerophospholipids to produce free fatty acids and lysophospholipids, and it can play a key function in lipid homeostasis, which is essential for membrane remodeling. *PLA2G6* is also linked to mitochondrial dynamics since high levels of ROS cause the accumulation of iPLA2-VI in mitochondria, which gives apoptosis protection [68]. In fact, the *Pla2g6* knockout mice are characterized by progressive cerebellar atrophy, Purkinje cell loss, and neuroinflammation, but no iron accumulation is observed. Moreover, this murine model displays degeneration of mitochondria and axonal termini in the spinal cord due to altered lipid profile, suggesting that the deficit of *Pla2g6* may lead to anomalies in membrane remodeling, and reduced mitochondrial potential in neurons, highlighting that *Pla2g6* participates in mitochondrial homeostasis. 

Mutations in *C19ORF12* are responsible for a relatively frequently NBIA form named MPAN (Mitochondrial membrane Protein-Associated Neurodegeneration, MIM 614298) (Table 1). The *C19ORF12* gene encodes for a transmembrane glycine zipper present into mitochondria, ER and MAMs (Mitochondria Associated Membranes) [69]. According to the findings described in fibroblasts from one MPAN patient, the loss of *C19ORF12* leads to high mitochondrial Ca^2+^ levels and increased H_2_O_2_ inducing oxidative damage [69]. The function of *C19ORF12* remains elusive, although a role as regulatory protein for magnesium transporters is suggested given its homology to the magnesium transporters E (MgtE) [69]. 

Two NBIA forms are due to iron-related genes, aceruloplasminemia (MIM 604290) and neuropherritinopathy (MIM 256600), caused by mutations in the *CP* and *FTL1* genes, respectively (Table 1). CP is responsible for the peroxidation of ferrous transferrin to ferric transferrin and due to its ferroxidase activity, is a strong antioxidant [70]. In cell systems, the absence of CP elicits that the ferrous iron entering the central nervous system cannot be oxidized and this is accumulated in cells as non-transferrin-bound iron that represents a toxic iron form, which induces oxidative stress, and in the end, causes neuronal death [71]. Several *Cp* knockout mice are available and no all of them have neurological phenotype; however, they share the abnormal iron accumulation and lipids peroxidation in the brain [72,73]. *FTL1* encodes the L-ferritin subunit, one of the two subunits of the ferritin, which is the major iron storage protein. The overexpression of mutated *FTL1* in cell models yields an increase of ferritin chains and a decrease of transferrin receptor 1 (TfR1) expression together with a production of ROS after treatment with H_2_O_2_, suggesting that the pathomechanism is likely due to deregulation of cellular iron homeostasis and oxidative damage, which would be primary cause of cell death [74,75]. Transgenic mice models, expressing mutated *Ftl1* show iron deposition and oxidative stress in brain, and a neurological deterioration with reduced lifespan and motor dysfunction [76,77,78].

The loss of *ATP13A2* function, which codifies an ATPase that participates in the maintaining of lysosomal iron stores, causes Kufor-Rakeb syndrome (MIM 606693) (Table 1) characterized by α-synuclein accumulation and mitochondrial stress-induced neurotoxicity [79,80]. Using Chinese Hamster Ovary (CHO) cells that stably express the ATP13A2 protein, the cytotoxicity induced by iron was reduced, which may suggest that the *ATP13A2* overexpression has a protective role against the cell damage caused by iron [81].

## 4. Neuromuscular Diseases

Charcot-Marie-Tooth (CMT) disease is one of the most clinically important inheritable peripheral neuropathies. CMT affects the structure and function of nerves, both motor and sensitive. There are at least 80 different genes affected in the disease. The genes affected may cause degeneration of either the axons or the myelin sheath in the peripheral nervous system. This degenerative process impairs action potential conduction, a decrement in the velocity of nerve transmission causing muscle atrophy and alterations in the sensitivity. It is currently incurable.

**Figure 3:**
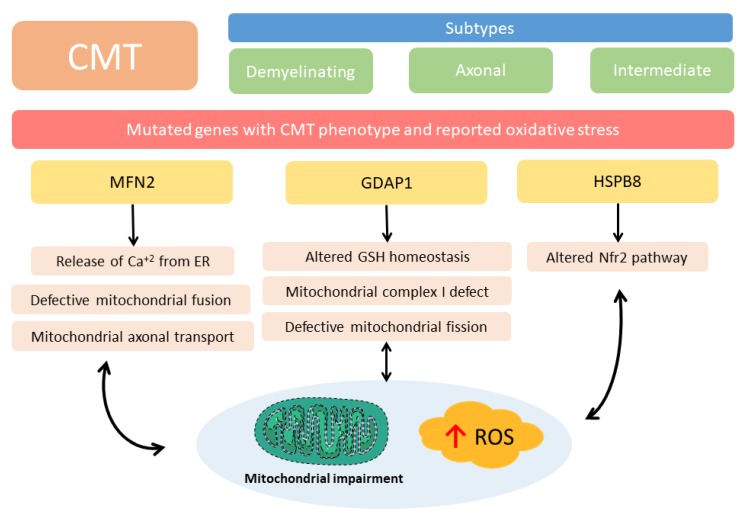
Charcot-Marie-Tooth disease (CMT) pathways related to oxidative stress. CMT is clinically divided into three main types, the demyelinating form, characterized by a decrease in the nervous conduction velocity; axonal CMT, where there is a loss in the number of axons and an intermediate clinical form with some decrease in the velocity of conduction and a decrease in the number of axons. At least 80 different genes are affected in the disease. Some of them produce oxidative stress or mitochondrial impairment. *MFN2* and *GDAP1* are the best known. *HSPB8* has been related to the Nrf2 pathway. ER: Endoplasmic reticulum, GDAP1: ganglioside induced differentiation associated protein 1, GSH: glutathione, HSPB8: Small Heat shock protein 8, MFN2: mitofusin 2, Nrf2: Nuclear factor erythroid 2-related factor 2, ROS: reactive oxygen species.

Classification of CMT is rather complex and is based on clinical and genetic criteria. CMT disease is clinically divided into three main types CMT1: the demyelinating form, characterized by a decrease in the nervous conduction velocity. CMT2 or axonal CMT, where there is a loss in the number of axons and a decrease in the number of Schwann cells; and an intermediate clinical form with some decrease in the velocity of conduction and a decrease in the number of axons. An important number of the mutated genes that produce CMT are related to mitochondrial proteins than develop an oxidative stress associated phenotype. However, not all the different types of CMT develop oxidative stress or mitochondrial impairment as far as we know. We will briefly review here the pathophysiological role of two of the most common causative genes for axonal CMT2, mitofusin 2 (*MFN2*) and ganglioside induced differentiation-associated protein 1 (*GDAP1*), where oxidative stress takes place when these proteins are mutated (Figure 3), but not all the other types of CMT where oxidative stress has not been demonstrated.

Mitofusin 2 protein is located mainly in the outer mitochondrial membrane where it contributes to the process of mitochondrial fusion by homo- and hetero-dimerization of MFN2 with mitofusin 1 (MFN1) [82]. MFN2 is also present in ER membrane-bound to mitochondrial MFN1 and MFN2, allowing the release of Ca^2+^ from ER into mitochondria [83,84]. MFN2 is also involved, possibly due to Ca^2+^ influx, in increasing outer membrane permeability, and consequently in the regulation of apoptosis [85]. It has been related to mitochondrial dynamics and in the control of the oxidative phosphorylation machinery [86]. MFN2 is present in many cell types. Nonetheless, the presence of *MFN2* mutations is only able to produce dysfunctions in peripheral nerves; this is the case of CMT. It seems that MFN1 is sufficient to accomplish the functions of MFN2 in the majority of tissues, except in peripheral nerves, due to the low physiological levels of MFN1 compared to other tissues [87]. It is still unclear why mutations in *MFN2* lead to CMT2A (MIM 609260) and what are the potential mechanisms that chief the pathological process, but clearly two are the most obvious: firstly, a defective mitochondrial fusion (for a review see [88]), and secondly, oxidative stress. In fact, Han et al. [89] have recently demonstrated in mouse adult hippocampus and cortex neurons that downregulation of MFN2 produces mitochondrial fragmentation and subsequent neurodegeneration induced by oxidative stress giving rise to neuroinflammation in vivo [89].

Among the main characteristics that are modified when *MFN2* is mutated, mitochondrial transport is of particular interest. An abnormal axonal transport of mitochondria could explain why mutations in *MFN2*, induce distal axonal degeneration giving rise to the clinical features of CMT2A neuropathy, where the longest axons are mostly affected. An important body of literature underscores the role of MFN2 in mitochondrial axonal transport (for a review see [87]).

GDAP1 is the other major protein mutated in CMT that gives rise to oxidative stress in CMT. It causes different clinical phenotypes of the disease (CMT2K and AR-CMT2K, MIM 607831; RI-CMTA, MIM 608340; CMT4A, MIM 214400). GDAP1 is ubiquitously expressed, but its presence is especially high in neurons [90]. GDAP1 is part of the glutathione S-transferase (GST) family [91], but it is unclear if the GST activity present in GDAP1 has any physiological relevance. However, inhibition of GDAP1 expression increases the susceptibility of the NSC34 motor neuron-like cells to GSH depletion [92]. Similarly, fibroblasts from CMT4A patients that also have reduced GDAP1 levels, show decreased GSH levels and mitochondrial membrane potential impairment, pointing oxidative stress as the main player in the pathogenesis of this type of CMT [92]. Very recently, it has been reported that mutations in the GST domain of GDAP1 are associated with a mitochondrial complex I defect and worsening of the clinical phenotype of the disease. This looks very interesting since other reported mutations that are located outside the GST domain show mild clinical phenotypes [93].

Decreased expression of GDAP1 levels in CMT produces mitochondrial morphological abnormalities impairing mitochondrial function, increasing the production of free radical species and altering the cytosolic calcium handling by mitochondria [94]. Decreased levels of GDAP1 produce disarrangement of Ca^2+^ metabolism in neurons and mitochondrial damage in a knockout mouse model of CMT. These mitochondrial changes give rise to axonal degeneration, neuronal death and neuroinflammation [95,96] producing a vicious circle of oxidative stress that contributes to the clinical features of the disease. However, we should remember that the correlation genotype/phenotype is complicated and CMT is a good example. In fact, Junctophilin-1 was described as a modifier gene of *GDAP1* and that this gives rise to phenotypic variability [97]. 

Missense mutations, such as p.K141N, in the heat shock protein HSPB8 cause CMT2L. According to Yang et al. [98], this mutation induced mitochondrial aggregation and caused increased oxidative stress injury in SH-SY5Y cells. L-3-n-butylphthalide (NBP), extracted from seeds of celery (Apium), was able to prevent mitochondrial aggregation and oxidative stress activating the Nrf2 pathway. Nrf2 is considered a master regulator that induces antioxidant gene expression through binding to antioxidant response elements (AREs) located in the promoters of genes encoding antioxidants enzymes. 

## 5. Inherited Retinal Dystrophies

Inherited retinal dystrophies (IRDs) are considered a group of heterogeneous retinal diseases that cause progressive and severe loss of vision by altering the retinal structure or/and retinal function [99]. IRDs are generally characterized by a permanent loss of light-sensitive retinal neurons, or their support cells leading eventually to legal blindness [99]. Although each disorder has individually a low prevalence, as a whole, they affect 1/3000–1/4000 individuals [100,101].

The features that best define IRDs are its high clinical variability and high genetic heterogeneity. Currently, 271 genes and 36 loci responsible for some type of IRD are known (RetNet, https://sph.uth.edu/retnet/; last accessed March 2020). Additionally, different mutations in the same gene may lead to different clinical conditions and to the same clinical condition but with different inheritance patterns [102]. 

Apart from inherited mutations, the retina is susceptible to several environmental insults and stress, including damage by light, and oxidative stress that can alter its structure and/or function [103]. The retina is especially vulnerable to changes in oxygen tension and oxidative stress because this is rich in polyunsaturated lipid membranes. Oxygen supply to the photoreceptors in the outer retina (main oxygen consumers) comes primarily from the choroid, while the retinal vasculature supplies oxygen to the inner retina. Elevated oxygen levels or hyperoxia are well known that results in photoreceptor cell death. Hyperoxia increases the production of ROS and RNS, which have been related to several IRD [104,105,106,107,108]. In particular, hyperoxia has been described in the outer retina of different models of Retinitis Pigmentosa (RP), the most prevalent IRD, such as RCS (Royal College of Surgeons) rats or P23H mutant transgenic rats [105,109]. Several studies support an oxygen toxicity hypothesis for RP. This hypothesis proposes that cone death (secondary photoreceptor loss) is due to hyperoxia, which is the consequence of the reduction in oxygen uptake following the initial loss of rod (due to various rod-related mutations) [110]. Some pieces of evidence of the gradual loss of oxygen metabolism in animal models of RP or patients include the altered distribution of intraretinal oxygen at different stages of the disease [109], the decreased content of oxygen-sensitive hypoxia-inducible factor-1α (HIF-1α) [111], or the thinning of inner retinal vessels [105,112,113]. Evidence for oxidative damage in RP are the increase of ROS or RNS, increase oxidation of macromolecules (lipids, proteins, and nucleic acids) and decrease in antioxidants in the retina, aqueous humor, and plasma of RP animal models and patients [107,114,115,116,117]. For instance, 8-oxo-7,8-dihydroguanine (8-oxoG), a major oxidized base in DNA, is increased in retinas of RP models and the vitreous of RP patients [118,119,120]. Accumulation of superoxide radicals and subsequent increase of peroxynitrite is present in the outer retinas of *rd1* mice, a model of RP, contributing to cone death [121]. Recently, Perdices et al. [122] also showed hepatic oxidative stress in P23H rhodopsin transgenic rats [105,122]. It is necessary to neutralize free radicals by the antioxidant defense system, otherwise, free radicals will interact with lipids, proteins, DNA, etc., affecting the cone survival. However, collected data from patients suggest that the antioxidant defense system is affected during RP [115,117,123]. 

Photoreceptors contain a high density of mitochondria in the inner segments that supply the ATP for the phototransduction processes, mainly through oxidative phosphorylation [124]. Most of the oxygen (~90%) is used within mitochondria. Then, mitochondrial oxygen metabolism is the main source of ROS production (e.g., superoxide and hydrogen peroxide) [125]. Mitochondrial oxidative stress and energy failure is a significant early event of IRD [126]. It was observed that retinal degeneration is accompanied by mitochondrial failure and metabolic imbalance, including downregulation of oxidative phosphorylation pathway and alterations in energetic pathways (glycolysis, β-oxidation), and changes in mitochondrial proteins [114,126,127,128].

When mitochondria are damaged, they can be selectively removed by autophagy, called mitophagy. Some genes associated with autosomal dominant RP, including *PRPF6*, *PRPF31*, *SNRNP200*, and *PRPF8* (core spliceosomal components) [129,130,131], are suggested to be responsible for ULK1 mRNA mis-splicing (an important protein for mitophagy initiation) and subsequent mitophagy defects [132]. 

Among genes involved in IRD, some mutations in mitochondrial genes are found such *IDH3A*, which generates NADH used for mitochondrial ATP production and is associated to early-onset retinal degeneration [133]; and aconitase (similar to *IDH3A*), that results in severe neurological disease apart from retinal degeneration [134,135]. 

The transcription factor Nrf2 exhibits antioxidant and anti-inflammatory properties in several tissues including the retina. Activation of Nrf2 through carnosic acid prevents retinal degeneration in Mef2d mice exposed to light damage [136]. Analogs of progesterone, which regulate Nrf2, also prevent retinal degeneration in inherited or light-damage models of retinal degeneration. For instance, Norgestrel activates and increases Nrf2 expression and its target, superoxide dismutase 2 reducing mitochondrial oxidative stress in a light damage model [137]. In RP, activation of Nrf2 and upregulation of its targets slow retinal degeneration in rabbits [138] and, mice by preserving photoreceptor, reducing inflammation and visual loss [139]. 

**Figure 4:**
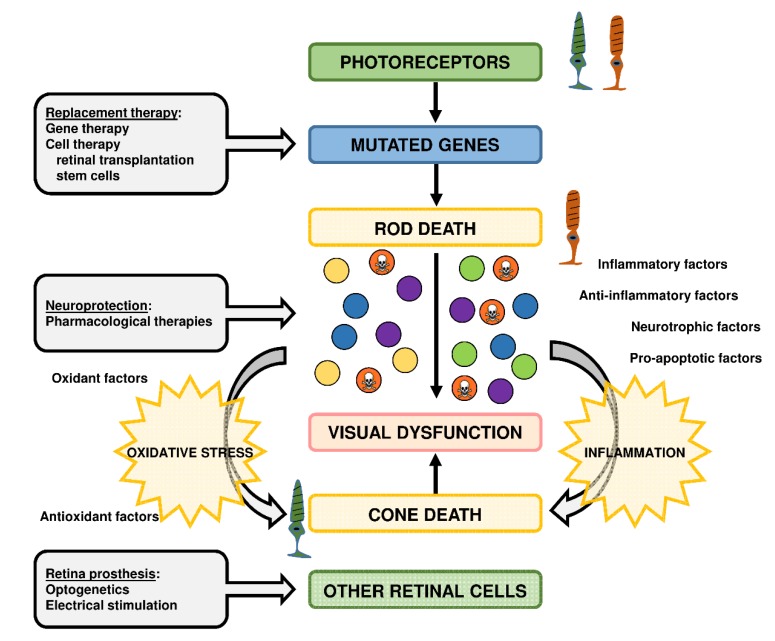
Therapeutic approaches for retinitis pigmentosa (RP), the most prevalent inherited retinal dystrophy. RP is characterized by progressive rod photoreceptor degeneration (mutated genes) in the initial stage and eventual cone photoreceptor degeneration in later stages. It is highly probable that cone degeneration is influenced by the release of inflammatory molecules, oxidant radicals, pro-apoptotic factors, etc. from rods and other cells, independently of the gene defect. Several therapeutic approaches have been developed to preserve retinal function or restore vision depending on the stage of the disease. They can be led to repair mutated photoreceptors (gene therapy), to replace damaged photoreceptors (cell therapy), or to protect photoreceptors and other retinal cells to the “toxic environment” (neuroprotective compounds including antioxidants). At later stages of RP, therapeutic approaches can be led to other retinal cells to restore visual activity with the remaining retinal circuits (optogenetics, retinal prostheses).

To date, there are no commercially available treatments to prevent photoreceptor cell loss and preserve vision in IRDs, but the development of several therapeutic approaches are ongoing, including nutritional supplements, neurotrophic factors, antioxidants supplementation, gene therapy, stem cell-based therapy, optogenetics, or retinal prostheses (Figure 4) [140,141,142,143]. These approaches depend on the subtype of IRD, its severity, and stage, the therapeutic target molecule, the knowledge of the mutated gene, etc. Some of these approaches try to correct or replace the genetic defect (gene or stem cell-based therapies) or to slow down the IRD progression (nutritional supplements, neurotrophic factors, antioxidants, etc.). In particular, several antioxidant formulations including curcumin, Tauroursodeoxycholic Acid (TUDCA), vitamin A, vitamin A in combination with docosahexaenoic acid (DHA), lutein, etc., have been used to mitigate the degenerative process in RP models and patients [116,144,145,146,147,148,149,150].

## 6. Rare Epilepsies

Epilepsy is a neurological disorder characterized by a predisposition to generate epileptic seizures and the associated cognitive, psychological, and social consequences [151]. The first-line treatment for epilepsy is anti-seizure drugs (ASDs). However, despite the availability of many ASDs, approximately one-third of patients fail to achieve seizure control or soon become resistant to the effects of the ASDs [151,152]. Consequently, there is a critical need for the development of innovative anti-epileptogenic treatment strategies to either ameliorate the progression or/and limit the detrimental consequences of the disease. Neuroinflammation, oxidative stress, and epilepsy are interconnected. It is becoming clear that brain inflammation promotes neuronal hyperexcitability and seizures, and that dysregulation in the glia immune-inflammatory function is a common factor that predisposes or contributes to the generation of seizures. At the same time, acute seizures upregulate the production of pro-inflammatory cytokines in microglia and astrocytes, triggering a downstream cascade of inflammatory mediators. Therefore, epileptic seizures, oxidative stress, and inflammatory mediators form a positive feedback loop, reinforcing each other [153]. It has also been shown that seizures can alter the redox state of the mitochondria [154]. As neuroinflammation and oxidative stress are intimately related hallmarks of many epileptic syndromes, this offers a window of opportunity for the use of anti-inflammatory and antioxidant drugs in these conditions. This possibility is supported by recent reports that clearly state that neuroinflammatory pathways may serve as treatment targets and biomarkers in different forms of epilepsy [153,155].

In this section, we will focus our attention on two different types of rare epilepsies: progressive myoclonus epilepsies (PMEs) and pediatric drug-resistant epilepsies. We describe examples of both types of pathology to illustrate the involvement of oxidative stress and neuroinflammation despite their clinical differences, and the window of opportunity that this information offers in the development of new treatments. 

### 6.1. Progressive Myoclonus Epilepsies (PMEs)

The PMEs are a group of neurological disorders characterized by the occurrence of focal and generalized seizures, myoclonus, and progressive neurological deterioration, accompanied by cerebellar signs and dementia [156,157,158,159,160]. PMEs consist of more than a dozen different diseases and they are classified as rare diseases since each of them affects less than 1/2000 individuals. The most common PME conditions are: (1) Unverricht–Lundborg disease (ULD) or epilepsy progressive myoclonus 1 (EPM1), due to mutations in the *CSTB* gene encoding cystatin B, a lysosomal cysteine protease inhibitor. (2) Lafora disease (LD) or epilepsy progressive myoclonus 2 (EPM2), due to mutations in either *EPM2A* gene, encoding the glucan phosphatase laforin, or *EPM2B* gene, encoding the E3-ubiquitin ligase malin. (3) Neuronal ceroid lipofuscinosis (NCL), a collection of disorders due to mutations in more than ten different *CLN* genes. (4) Sialidosis, a lysosomal storage disease due to mutations in the *NEU1* gene encoding the lysosomal enzyme alpha-*N*-acetylneuraminidase; and (5) mitochondrial myopathies, such as myoclonic epilepsy with ragged fibers (MERRF) due to mutations in the mitochondrial gene *MT-TK* encoding the tRNA^Lys^, among others [158].

Although the primary cause of PMEs is different in each case, recent reports suggest that oxidative stress and neuroinflammation are common traits in all these conditions [161,162,163]. This reinforces the idea that oxidative stress and neuroinflammation are at the crossroad of rare neurodegenerative disorders. Among PMEs, we will focus on the two most characterized forms: ULD and LD.

#### 6.1.1. Unverricht–Lundborg Disease (ULD)

The onset of ULD (MIM 254800) is around late childhood and early adolescence. It is characterized by the appearance of generalized tonic-clonic seizures that may occur without prior myoclonic jerks. As the disease progresses, myoclonus increases in intensity and frequency culminating in generalized tonic-clonic seizures. ULD also progresses with associated neurological symptoms, such as ataxia, impaired walking, and cognitive impairment. On the contrary, to other PMEs, early death is not common in ULD and the outcome of adult patients ranges from minimal impairment with an independent active life to severe disability [164,165]. ULD is an autosomal recessive disorder due to mutations in the gene encoding cystatin B/Stefin B (*CSTB*), an 11 kDa lysosomal cysteine protease inhibitor. The most common mutation leads to the expansion of minisatellite sequence repeats (CCCCGCCCCGCG) in the 5′-untranslated region of the *CSTB* gene, to reach a number of 30–80 repeats in the causative disease range, leading to its reduced expression [165]. In addition, frameshift mutations and deletions are also found among ULD patients, although they are less common [164].

There is a mouse model of ULD that lacks the *CSTB* gene (*Cstb*^−/−^), which reproduces the pathophysiology of the disease: they show myoclonic seizures, ataxia, and progressive neuronal loss. In these mice, it has been proposed that early-onset neuroinflammation was key in the pathogenesis of ULD and that glial-derived pro-inflammatory chemokines and cytokines contributed to recurrent excitation and epilepsy [161]. In the absence of *Cstb*, there is also a clear mitochondrial dysfunction, with decreased membrane potential and increased ROS production, which could stimulate an inflammatory response. The authors suggest that this mitochondrial dysfunction could be the primary cause of the pathophysiology of ULD [166].

#### 6.1.2. Lafora Disease (LD)

**Figure 5:**
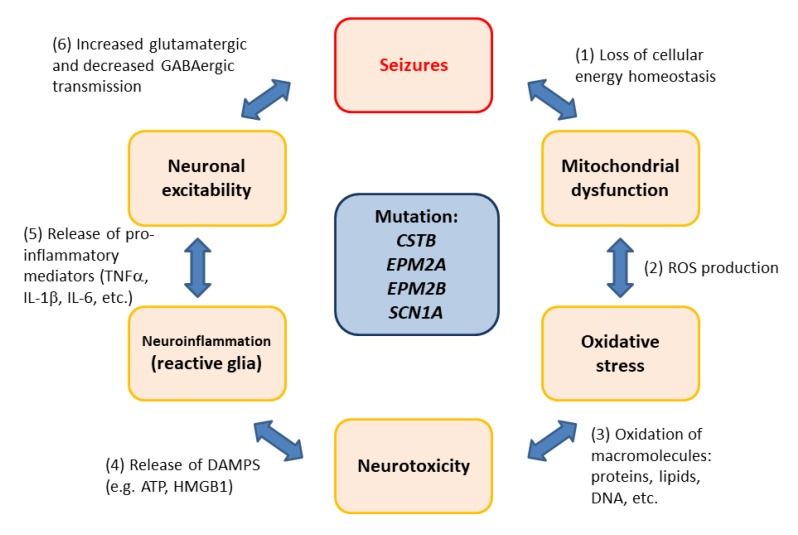
The vicious circle of oxidative stress and neuroinflammation in rare epilepsies with a genetic origin. (1) There is an inverse relationship between mitochondria functionality and the appearance of seizures. Alterations in cellular energy homeostasis result in neuronal pathology. (2) Mitochondrial dysfunction leads to oxidative stress due to the accumulation of reactive oxygen species (ROS). (3) Oxidized macromolecules (e.g., proteins, lipids, DNA) affect neuronal viability, leading to neurotoxicity. (4) Neuronal release of danger associated molecular patterns (DAMPs; e.g., HMGB1, ATP) triggers a neuroinflammatory response, leading to the activation of glia (microglia and astrocytes). (5) The production of pro-inflammatory mediators (TNFα, IL-1β, IL-6, etc.) affects neuronal excitability. (6) Increased glutamatergic transmission and decreased GABAergic inhibition leads to seizures.

LD (MIM 254780) is a rare, fatal form of PME characterized by the accumulation of insoluble poorly branched glycogen inclusions (named Lafora bodies, LBs) in the brain and peripheral tissues. The onset of LD occurs around late childhood and early adolescence and it is characterized by the appearance of generalized tonic-clonic seizures, myoclonus, absences, and visual hallucinations. The disease progresses rapidly with a worsening of seizures and dementia, leading to the death of the patient after a decade from the onset of the first symptoms [167]. LD is caused by mutations in the *EPM2A* gene, which encodes the glucan phosphatase laforin [168], and the *EPM2B* gene, encoding the E3-ubiquitin ligase malin [169]. Laforin and malin form a functional complex that regulates glycogen synthesis, the homeostasis of glucose transporters, the maintenance of proteostasis and the response to oxidative stress, among other physiological pathways [167] (Figure 5).

LBs contain, in addition to carbohydrate moieties, up to 6% of proteinaceous material, which includes glycogen metabolizing enzymes, laforin, chaperones, autophagic components, proteasome subunits, advanced glycation end products, etc. [170,171,172]. Most of these products are post-translationally modified by ubiquitination, suggesting a cellular attempt to eliminate LBs by the usual protein clearance mechanisms, namely proteasomes and autophagy. However, these mechanisms are unsuccessful in disposing of the LBs and become deteriorated. In fact, it has been suggested that in the absence of laforin or malin there is a decrease in the activity of proteasomes [173,174] and an impairment in autophagy, which likely occurs at the initial step of autophagosome formation [134,170,175,176]. As a consequence, activation of the unfolded protein response pathway proceeds, which eventually leads to increased endoplasmic reticulum stress [171,174]. In this regard, it has also been described that laforin interacts physically with the chaperone Hsp70 and helps to decrease the toxicity of the unfolded proteins [177]. Therefore, all these results suggest a possible role of the laforin-malin complex in regulating cellular proteostasis [134,170,173,174,175,176].

Since the autophagy machinery is impaired, mechanisms that require this process are also affected in cellular and animal models of the disease. In this sense, we have recently described a defect in mitophagy, most likely due to defects in the initial steps of autophagosome formation [178]. Finally, we have also reported that oxidative stress is affected in the absence of a functional laforin-malin complex [179]. We demonstrated that in cellular and animal models of LD, there were higher levels of ROS and oxidative stress products. This defect was probably due to an altered mitochondrial function and also, to alterations in the levels of antioxidant enzymes and to decreased activity of enzymes involved in the detoxification of ROS [179].

Recently, we have analyzed by RNA-Seq technology the genes that were differentially expressed in the brain of *Epm2a*^−/−^ and *Epm2b*^−/−^ mice in comparison to control animals and observed that *Epm2a*^−/−^ and *Epm2*^−/−^ mouse brains overexpress a common set of genes mostly related to inflammation [162]. We also defined that reactive glia was responsible for the expression of these genes. These results are in agreement with the presence of pro-inflammatory markers (e.g., IL-1beta, TNF-alpha, and IL-6) in the brain of *Epm2a*^−/−^ and *Epm2*^−/−^ mice [180], and suggest that neuroinflammation should be considered as one of the most important traits in LD.

### 6.2. Dravet Syndrome

Pediatric drug-resistant epilepsies include a group of epileptic encephalopathies that are characterized by being caused by monogenic mutations, an early onset before or around the first year of age, the presence of associated developmental and cognitive impairments and resistance to common anticonvulsant treatments [181]. These pathologies include the Otahara, West, atypical Rett, and Dravet syndrome (DS) or severe myoclonic epilepsy of infancy (SMEI). The DS (MIM 607208) can be considered as a paradigmatic example in this type of epilepsy, since the involved gene was one of the first to be identified, and has well-known genetics, etiology, and pathophysiology [182,183,184]. DS has an early onset in the first few months of life, usually triggered by fever, with a clonic or hemiclonic seizure, which is followed by further episodes that can be prolonged and even result in status epilepticus. With time, patients usually suffer more febrile or afebrile seizures that can be of different types from the initial ones: tonic-clonic, myoclonic, absence. In addition to seizures, they can also suffer cognitive delay, movement disorders and, more seriously, sudden death in a relatively high proportion (>10%). 

When the genetic basis of DS was uncovered [185], it came as a surprise that it was caused by dominant mutations on the *SCN1A* gene. This gene codes for the α subunit of the type 1 voltage-gated sodium channel, and these channels are usually involved in the excitation of neuronal and muscular cells. The conundrum was resolved when it was found that the mutations prevented the activation of GABAergic inhibitory interneurons, not the excitatory neurons, disrupting the excitation/inhibition balance [186,187]. There are no efficient medications to control the seizures and the neurological damage, although some first-line ASDs such as valproate, stiripentol, or benzodiazepines can help to control the seizures [188]; the most recent therapeutic development has been the successful clinical trials of cannabidiol (CBD) [189], which was approved to treat DS in America and Europe in 2019. 

In patients suffering from severe DS, there are evident features of encephalopathy [190,191], probably due to an exacerbated inflammatory state. Although oxidative stress is not the primary cause of DS, the available evidence strongly indicates that oxidative stress and neuroinflammation are involved in the pathology pediatric epilepsy, mainly in the ensuing encephalopathy and deterioration of cognitive capabilities. Nuclear Metabolic Resonance (NMR) metabolomic analysis of pediatric drug-resistant epilepsies revealed changes in lactate, creatine, citrate, and lipids consistent with an increase in oxidative stress [192]. Regarding DS, GABAergic neurons differentiated from patient-derived iPSCs displayed a defective response to oxidative stress [193]. In this study, the authors also found reduced levels of the *GSTM1* gene, which is also associated with epilepsy and encodes a glutathione-S-transferase, partly explaining the susceptibility to oxidative stress. 

### 6.3. Oxidative Stress and Neuroinflammation as Therapeutic Targets in Rare Epilepsies

Neuroinflammation and oxidative stress emerge as important traits to be considered in the pathophysiology of rare epilepsies, and therefore, they constitute pharmacological targets for this group of diseases. We could hypothesize that mitochondria dysfunction accompanied by ROS production could be the trigger of the initial inflammatory response, which will eventually lead to epilepsy.

Regarding oxidative stress, some of the current treatments could already be addressing this problem. In addition to DS, cannabidiol (CBD) is being used or in clinical trials for several types of epilepsy [194], and it has a neuroprotective effect by inhibiting nitric oxide production, one of the main neurotoxic effectors [195]. The ketogenic diet, high in fats and low in carbohydrates and proteins, is also widely used to treat refractory epilepsies [196]. The β-oxidation of fatty acids produces ketone bodies, acetoacetate, and β-hydroxybutyrate, a metabolic state similar to starvation known as ketosis. In a rat model of fever induced by lipopolysaccharides, the ketogenic diet produced an anti-inflammatory state, with reduced levels of pro-inflammatory cytokines [197]. β-hydroxybutyrate acts as an epigenetic factor through histone deacetylases and increases the expression of the antioxidant genes *FOXO3A* and *MT2* [198].

The initial oxidative insult could activate astrocytes and/or microglia to prime a general activation of these cells in the brain, which would then lead to neuronal degeneration. This is an important issue since it enhances the importance of astrocytes and microglia in this pathology and identifies these cells as the primary cause of the disease instead of the neurocentric idea that they are produced by initial neuronal problems. In addition, it has been demonstrated that anti-inflammatory interventions in animal models of epilepsy have both anti-epileptogenic and disease-modifying therapeutic effects [153,155]. However, it has also been stated that general anti-inflammatory drugs should not be used due to their wide central and peripheral effects [199] and that the anti-inflammatory strategy should be based on the signaling pathways that are altered in each epileptic condition. Some of these specific compounds are already in clinical use for the treatment of autoimmune diseases, so the use of specific brain-penetrant anti-inflammatory compounds that are used in other pathologies could be repurposed for drug-resistant epilepsies [153]. Therefore, the use of specific anti-inflammatory compounds is an alternative therapeutic strategy that should be explored for the treatment of PMEs.

## 7. Conclusions

As it is well known, free radical species are physiological cell signaling molecules, but when there is an imbalance in their levels, pathological oxidative stress can induce paramount changes in cell function even inducing cell death. In this scenario, the role of mitochondria, one of the main cellular sources of ROS, plays a pivotal role. The division between cause and effect is not always easy to establish because mitochondrial dysfunction and oxidative stress are tightly dependent on each other in the pathogenesis of neurodegenerative disorders. Moreover, oxidative stress and inflammation feed on each other and contribute to neurodegeneration. The NADPH oxidase and hydrogen peroxide release from activated inflammatory cells are also important factors. Here, we have reviewed the disease mechanism in disparate groups of neurodegenerative rare disorders that comprise retinopathies, epilepsies, movement disorders, and neuromuscular diseases. All of them are monogenic rare disorders that share oxidative stress as a common factor and may represent potential targets for therapies. Many neurodegenerative polygenic diseases (e.g., Alzheimer’s disease, amyotrophic lateral sclerosis, Parkinson’s disease, etc.) also show oxidative stress-related damage. Therefore, to unravel the disease mechanisms of Mendelian conditions results of great interest because this should be easier than for common disorders, and the findings may be also applied in the understanding of the pathophysiological mechanisms underlying common entities. 

## Figures and Tables

**Table 1:** NBIA forms: genes and pathways.

NBIA form	MIM #	Frequency Inheritance	Gene MIM *	Protein Location	Pathway
Pantothenate kinase-associated neurodegeneration (PKAN)	234200	35%–50% AR	*PANK2* 606157	Mitochondria	CoA synthesis (fatty acid metabolism)
Phospholipase 2, group VI-associated neurodegeneration (PLAN)	610217	20% AR	*PLA2G6* 603604	Mitochondria, ER, cytosol	Membrane phospholipids turnover
Mitochondrial membrane protein-associated neurodegeneration (MPAN)	614298	6%–10% AR	*C19ORF12* 614297	Mitochondria, ER, MAM	Lipid metabolism ? Membrane remodeling ?
β-propeller-associated neurodegeneration (BPAN)	300894	1%–2% XD	*WDR45* 300526	ER	Autophagy
Fatty acid hydroxylase-associated neurodegeneration (FA2H)	612319	Rare AR	*FA2H* 611026	ER	Lipid metabolism Membrane remodeling
Neuroferritinopathy (NF)	606159	Rare AD	*FTL1* 134790	Cytosol	Iron homeostasis
Aceruloplasminemia	604290	Rare AR	*CP* 117700	Plasma membrane	Iron homeostasis
Woodhouse-Sakati syndrome	241080	Rare AR	*DCAF17* 612515	Nucleolus	Unknown
Kufor-Rakeb syndrome	606693	2 probands AR	*ATP13A2* 610513	Lysosome, mitochondria	Autophagy
COASY protein-associated neurodegeneration (CoPAN)	615643	4 probands AR	*COASY* 609855	Mitochondria, cytosol	CoA synthesis (fatty acid metabolism)
Jaberi-Elahi syndrome (JABELS) + NBIA	617988	1 family AR	*GTPBP2* 607434	Cytoplasm	Unknown
Leukoencephalopathy with dystonia and motor neuropathy + NBIA	613724	1 proband AR	*SCP2* 184755	Peroxisomes	Lipid metabolism Membrane remodeling
NBIA7	617916	1 family AR	*REPS1* 614825	Cytoplasm, endosome	Endocytosis Vesicle transport
Hereditary spastic paraplegia + NBIA	612936	1 family AR	*AP4M1* 602296	Endosome	Vesicle formation
NBIA8	617917	1 proband AR	*CRAT* 600184	Mitochondria	Lipid metabolism

MIM: Mendelian Inheritance in Man database, # refer to diseases, * refer to genes, ? refer to not known with absolute certainty; NBIA: Neurodegeneration with brain iron accumulation; MIM: Mendelian inheritance in man; AR: autosomal recessive; XD: X-linked dominant; AD: autosomal dominant; ER: endoplasmic reticulum; MAM: Mitochondrial-associated membrane.
